# How sweet it is – making the most of carbohydrate metabolism

**DOI:** 10.1186/1753-6561-5-S7-I16

**Published:** 2011-09-13

**Authors:** Shawn Mansfield

**Affiliations:** 1 Department of Wood Science, University of British Columbia, Vancouver, Canada

## 

Like all plants, trees constantly monitor endogenous and environmental cues, and use this information to make adjustments in resource allocation, both to maximize resource acquisition and to minimize exposure to deleterious phenomena. To accomplish this, trees possess mechanisms that integrate and interpret the information provided by internal and environmental signals. Ultimately, these phenomena are controlled at the level of gene expression, which consequently translates into the accumulation of a variety of soluble metabolites, including carbohydrates. Trees are relatively “plastic” in their ability to allocate resources, and such plasticity has an adaptive significance in that it allows trees to (i) match resource allocation with resource acquisition, (ii) acquire new resources more effectively, and in some instances (iii) avoid adverse conditions. The inherent plasticity in resource acquisition can have a profound effect not only on the development and physiology of trees, but also on the industrial harvesting and utilization of the terminal lignocellulosic resource.

Photosynthetic carbon capture by trees represents a major sink for atmospheric CO_2_, ultimately terminating in the synthesis of a secondary plant cell wall – a complex matrix of polysaccharides intricately linked to lignin. Therefore, engineering plant where resource allocation is directed to vegetative biomass and fibre properties should have an effect on the plant cell wall traits. As such we have targeted the biosynthetic processes governing sucrose metabolism by mis-regulation of key enzymes associated with the catabolism and anabolism of sucrose in attempts cellulose production – both quantity and quality. This paper will discuss the results of these efforts, and illustrate the altered performance of such plants for industrial use.

